# A review of the pharmacological action and mechanism of natural plant polysaccharides in depression

**DOI:** 10.3389/fphar.2024.1348019

**Published:** 2024-02-08

**Authors:** Yu-He Yang, Chen-Xue Li, Ruo-Bing Zhang, Ying Shen, Xue-Jiao Xu, Qin-Ming Yu

**Affiliations:** ^1^ Heilongjiang University of Chinese Medicine, Harbin, China; ^2^ Harbin University of Commerce, Harbin, China

**Keywords:** polysaccharides, natural plant, depression, pharmacological action, mechanism

## Abstract

Depression is a prevalent mental disorder. However, clinical treatment options primarily based on chemical drugs have demonstrated varying degrees of adverse reactions and drug resistance, including somnolence, nausea, and cognitive impairment. Therefore, the development of novel antidepressant medications that effectively reduce suffering and side effects has become a prominent area of research. Polysaccharides are bioactive compounds extracted from natural plants that possess diverse pharmacological activities and medicinal values. It has been discovered that polysaccharides can effectively mitigate depression symptoms. This paper provides an overview of the pharmacological action and mechanisms, intervention approaches, and experimental models regarding the antidepressant effects of polysaccharides derived from various natural sources. Additionally, we summarize the roles and potential mechanisms through which these polysaccharides prevent depression by regulating neurotransmitters, HPA axis, neurotrophic factors, neuroinflammation, oxidative stress, tryptophan metabolism, and gut microbiota. Natural plant polysaccharides hold promise as adjunctive antidepressants for prevention, reduction, and treatment of depression by exerting their therapeutic effects through multiple pathways and targets. Therefore, this review aims to provide scientific evidence for developing polysaccharide resources as effective antidepressant drugs.

## 1 Introduction

**FIGURE 1:**
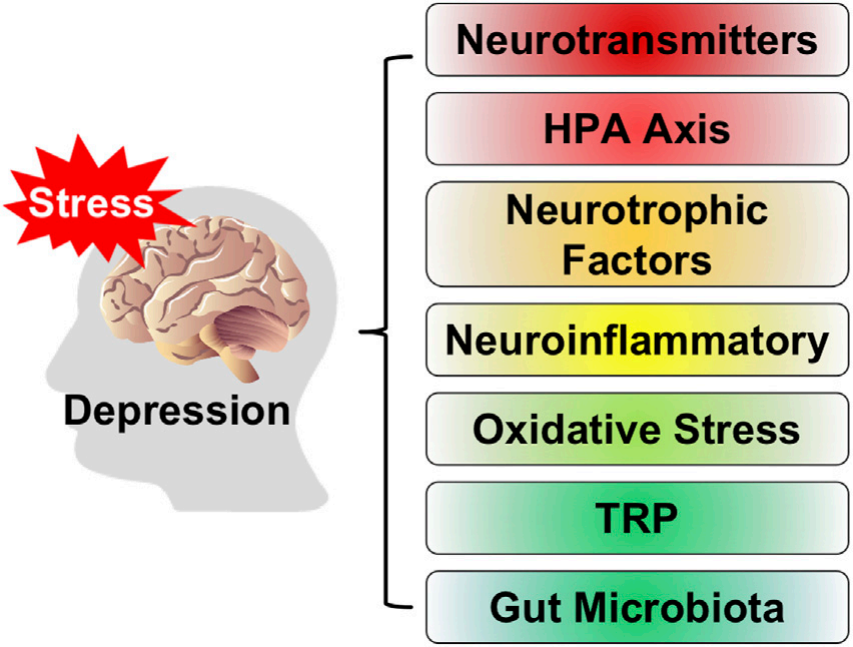
The pathogenesis of depression.

Depression, as a common mental disorder, seriously endangers human physical and mental health (Paykel, 2008). People with depression usually show symptoms such as lack of interest, slow thinking, low mood, loss of appetite, insomnia and sleeplessness, and decreased willpower (Thapar et al., 2022). With socio-economic development and incremental pressure on people, the number of people suffering from depression has increased dramatically, bringing serious costs to society and families. Because the severe illness is often accompanied by self-harm, suicidal tendencies and violent tendencies, making depression become the disease with the highest suicide rate in the world. At the same time, the disease can seriously affect or even damage the patient’s digestive system, immune system, and nervous system (Fries et al., 2023). The pathogenesis of depression is complex (Guo et al., 2023), and its pathogenic mechanism is not yet clear. Existing studies have shown that the main features of depression are abnormal neurotransmitter levels in patients, abnormal function of the hypothalamic-pituitary-adrenal (HPA) axis, hormonal imbalance in the organism with inflammation and oxidative stress, neurotrophic factors, tryptophan metabolism, and the occurrence of gut microbiota-related diseases, as shown in Figure 1.

At present, depression is mostly treated with chemical drugs, antipsychotic drugs, which can be divided into first- and second-generation drugs (Rybakowski, 2023). The first-generation drugs are mainly dopamine receptor blockers, which have antipsychotic effects by blocking dopamine receptors in the dopamine pathway of the central nervous system. And their long-term use can cause side effects such as drowsiness and cardiac rhythm disturbances. In response to the problems caused by the first-generation drugs, the second generation of drugs represented by fluoxetine and paroxetine combines therapeutic efficacy and low side effects, which improves depression and relieve anxiety though inhibiting the reuptake of 5-serotonin (5-HT) (Grinchii and Dremencov, 2020). 5-HT, as one of the indole derivatives, is a neurotransmitter with the effects on mood regulation, memory improvement and cognitive enhancement that exerts inhibitory effects in the neuroendocrine system, which is considered to be a messenger of pleasure (Moncrieff et al., 2022). The reduction of its levels is closely correlated with the occurrence of depression and anxiety. However, second-generation drugs tend to have slow onset of action, weak anxiolytic effects and are associated with prolonged cognitive impairment (Barbuti et al., 2023). While these drugs can relieve the symptoms of depression, there is currently no cure for depression. At the same time, antipsychotic drugs are limited by the fact that they can only act at one site or one type of target site. As a result, depression is characterized by low remission rate, high recurrence rate and significant side effects for patients, and only 12.7% of patients received minimal adequate treatment (Grinchii and Dremencov, 2020). Despite the clear target and rapid efficacy of Western clinical antidepressant drugs, they also have the disadvantages of low efficiency, single pathway, and serious adverse effects. Due to the complexity of the pathogenesis of depression, single-target drugs have limited amelioration of depression and serious side effects, the exploitation of new multi-target drugs for the treatment of depression is particularly important.

Therefore, the development of an antidepressant drug, with high efficiency, long-term use, and low side effects, has become a recent research hotspot. Polysaccharides from natural sources have received widespread attention due to their wide range of pharmacological activities and fewer side effects (Song et al., 2021). Polysaccharides are the biologically active macromolecules formed by monosaccharides linked by glycosidic bonds, which are formed by polyhydroxy polymers and their derivatives. They have widespread in natural products of higher plants, animals, microorganisms and algae. Numerous studies have shown that polysaccharides have anti-inflammatory, antioxidant, antiviral, immune regulation, hypoglycemic and hypolipidemic effects, and regulation of gut microbiota (Mu et al., 2021; Ji et al., 2023). Meanwhile, existing studies have demonstrated that the polysaccharide molecules in natural products can effectively alleviate depression, and their mechanism of action has attracted extensive attention from researchers (Yi et al., 2009; Song et al., 2022; Guo et al., 2023). The progress has been reported in the study of the multi-targeted therapeutic effects of polysaccharides in depression. Some researchers have pointed out that the mechanism is achieved through the modulation of brain function, biological and immune barriers. The polysaccharide molecules in natural products can usually be used in multiple targets with significant therapeutic effects and fewer side effects (Wang et al., 2022b). However, there are fewer reports on the relationship between polysaccharides and depression. This review summarizes the recent progress of research on the antidepressant effects of polysaccharides by intervening in different mechanisms in recent years, providing references for further research on the prevention, alleviation and treatment of depression and the development of therapeutic drugs.

## 2 Antidepressant mechanism of natural plant polysaccharides

Polysaccharides in natural plant sources alleviate depression by regulating the expression of neurotransmitters and their receptors, HPA axis, neurotrophic factors, neuroinflammatory, oxidative stress, tryptophan metabolism and gut microbiota. The various plant polysaccharides from different sources can induce antidepressant effects through diverse mechanisms of action, as summarized in Table 1.

**TABLE 1:** Validation models, targeting mechanisms and intervention results of natural plant polysaccharides.

Name	Polysaccharide source	Monosaccharides composition	Validation model	Administration	Target mechanism	Effect	Positive control	Ref.
PSP	*Polygonatum sibiricum* Red.	Ara: Glc: GlcA: Gal: GalA: Man: Rha: Rib = 13.7: 82.9: 3.7: 36.2: 4.3: 52.5: 3.3: 1.0	CUMS-induced ICR mice;	100–400 mg/kg	Target neurotransmitters;	↑: 5-HT, DA, NE, ERK1/2, NF-κB, GFAP, Calpastatin, PTEN, SCOP, Nrf2	Fluoxetine;	Shen et al., 2021 (2022), Wei et al. (2022)
			LPS and CUMS-induced C57BL/6 mice;		Target HPA;	↓: TNF-α, IL-10, TRP, 3-HK, CORT, Caspase-3, GluN2A, GluN2B, Calpain-1, NLRP3, ASC, Iba1, P-ERK, Caspase-1, Cleaved-caspase-1	Calpeptin;	
			LPS-induced HT-22 cells;		Target neuroinflammation;		MCC	
			CUMS-induced BALB/c mice		Target TRP metabolism;			
					Target gut microbiota			
YLSP	*Millettia pulchra* (Benth.) Kurz var. *Laxior* (Dunn) Z. Wei	Ara: Glc = 90.79%:9.21%	UCMS-induced KM mice	150–600 mg/kg; 1,200 mg/kg	Target neurotransmitters;	↑: NE, DA, 5-HT, cAMP, BDNF	Fluoxetine	Liang et al., 2010 (2012), Lu et al. (2013)
					Target NTF			
ASP	*Angelica sinensis* (Oliv.) Diels	NA	CUMS-induced C57BL/6 mice	20 and 40 mg/kg	Target neurotransmitters	↑: TPH1, 5-HT, DA, GABA/GLU	Fluoxetine	Ding et al. (2021)
SP	*Poria cocos* (Schw.) Wolf	NA	Ovariectomy and CUMS-induced Sprague-Dawley rats	25, 50, and 100 mg/kg	Target neurotransmitters;	↑: CREB, BDNF, GluR1, P-GluR1	NA	Zhang et al. (2019), Zhou and Li (2020)
					Target neuroinflammation			
PCAP	*Poria cocos* (Schw.) Wolf	NA	CUMS-induced SD rats	0.1, 0.3, and 0.5 g/kg/d	Target NTF;	↑: BDNF, 5-HT, 5-HIAA, DA, NE, pro-caspase-1, pro-IL-1β, pro-IL-18	Fluoxetine	Chen et al. (2021)
					Target neuroinflammation	↓: GLU, IL-1β, IL-18, TNF-α, ASC, caspase-1, IL-1β, IL-18, NLRP3		
LNT	*Lentinus edodes* (Berk.) Sing.	NA	CUMS-induced KM mice	2.5 and 5.0 mg/kg	Target oxidative	↑: 5-HT1A, SOD	NA	Ma et al. (2015)
						↓: MDA, TNF-α, IL-6		
GLP	*Ganoderma lucidum* (Leyss.ex Fr.) Karst.	Rha: Xyl: Fru: Gal: Man: Glu = 0.793: 0.964: 2.944: 0.167: 0.384: 7.94	CSDS-induced C57BL/6 mice	1, 5, and 12.5 mg/kg/d	Target neurotransmitters;	↓: IL-1β, TNF-α	Imipramine	Cai et al. (2017), Li et al. (2021)
					Target NTF;	↑: IL-10, BDNF, GluA1 S845, GluA1, GluA2		
					Target neuroinflammation			
LBP	*Lycium barbarum* L.	Rha: Ara: Xyl: Man: Glc: Gal: GalA = 1: 8.34: 1.25: 1.26: 1.91: 7.05: 15.28	PTSD-induced SD rats;	25, 50, and 100 mg/kg/d for 3 weeks;	Target neurotransmitters;	↓: NR2B-Ca MKII, serum CORT, LPO, Bcl-2, PARP	Paroxetine;	Chu, 2019; Zhao et al. (2019), Fu et al. (2021)
			AS-induced C57BL/6 mice;	80 mg/kg/d for 4 weeks;	Target oxidative		Amitriptyline	
			Reserpine-induced C57BL/6 mice	5 mg/kg for 28 days				
LLP	*Lilium lancifolium* Thunb.	Man: GlcA: NAG: Glc: Gal: Fuc = 1: 0.19: 0.32: 0.46: 0.57: 0.25	CUMS-induced KM mice	0.2 g/kg/d	Target neurotransmitters;	↑: 5-HT, ADCY6, PKA, CREB-1, BDNF	Fluoxetine	Liu et al. (2022b)
					Target HPA;	↓: ACTH, CORT		
					Target NTF;			
APS	*Astragalus membranaceus* (Fisch.) Bge. var. *mongholicus* (Bge.) Hsiao	Man: Rha: GlcUA: GalUA: Glc: Gal = 29.12: 1.89: 4.00: 1.35 : 1: 81.97	CUMS-induced KM mice;	200 and 400 mg/kg/d for 4 weeks;	Target neurotransmitters;	↑: ADCY6, PKA, CREB-1, BDNF, Nrf2, HO-1, SOD, CAT, GSH-Px	Fluoxetine	Wang et al., 2018 (2019a), Liu et al., 2019a (2022b), Su et al. (2021)
			CUMS-induced Wistar rats;	0.2 g/kg/day	Target HPA;	↓: MCAO, MDA; TNF-α, IL-1β, IL-6, NF-κB		
			PSD-induced Wistar rats		Target NTF;			
					Target neuroinflammation;			
					Target oxidative			
DOP	*Dendrobium officinale* Kimura et Migo	rhamnose, arabinose, fucose, mannose and glucose, and glycosidic	Ovariectomy and CUMS-induced KM mice;	50, 150, 300, and 600 mg/kg	Target HPA;	↓: BDNF–TrkB–CREB, CRH, ACTH, serum CORT	Fluoxetine	Yang et al. (2022), Zhang et al. (2022)
			CUMS-induced SD rats		Target NTF;			
					Target gut microbiota			
GEP	*Gastrodia elata* Bl.	NA	LPS-induced C57BL/6 mice	50, 100, and 200 mg/kg	Target neuroinflammation	↓: TNF-α, IL-1β	Fluoxetine	Liu et al. (2021)
MCP	*Momordica charantia* L.	NA	CSDS-induced C57 mice	100, 200, and 400 mg/kg/d	Target neuroinflammation	↓: TNF-α, IL-6, IL-1β, JNK3, c-Jun, P-110β proteins, JNK3/PI3K/AKT	NA	Deng et al. (2019)
OP	*Abelmoschus esculentus* (L.) Moecnch	GalA, GlcA, Gal, Ara, Rha = 42.01%, 39.25%, 7.12%、5.51%, 6.11%	CUMS-induced C57BL/6 mice;	30 μg	Target neuroinflammation;	↓: TLR4/NF-κB, MAPKs, MAPKs, NO, TNF-α, IL-6, IL-1β	Isotype control antibodies or goat IgG	Brancato et al. (2013), Yan et al. (2020)
			LPS-induced BV2 cells		Target gut microbiota	↑: acetic acid, propionic acid, and butyric acid		
LJP	*Lonicera japonica* Thunb.	GalA: Rha: Gal: Ara: Glc: Man = 8.7%: 8.2%: 16.2%: 19.5%: 26.9%: 20.5%	CUMS-induced KM mice	100 mg/kg	Target neuroinflammation	↓: NLRP3, IL-1β, caspase-1	Fluoxetine	Liu et al. (2019b)
APSP	*Acanthopanax senticosus* (Rupr.etMaxim.) Harms	NA	CUMS-induced Wistar rats	60, 120 mg/kg	Target neuroinflammation;	↓: IL-1β, IL-6, TNF-α, MDA	Fluoxetine hydrochloride group	Ding et al. (2022)
					Target oxidative	↑: CAT, SOD, p-PI3K, p-Akt, p-mTOR		
GBP	*Ginkgo biloba* L.	mannose, rhamnose, glucuronic acid, galactose, arabinose	UCMS-induced BALB/c mice	300 mg/kg	Target gut microbiota	↑: 5-HT, DA, *Lactobacillus*	Paroxetine	Chen et al. (2019)
TG	*Cistanche tubulosa* (Schenk) Wight	NA	CUMS-induced SD rats	0.26 g/kg	Target TRP metabolism;	↑: TRP	Imipramine;	Fan et al. (2021)
					Target gut microbiota	↓: KYN/TRP, IDO1	Fluoxetine	
DPR	*Porphyra haitanensis* Chang et Zheng	NA	LPS-induced C57BL/6 mice	100, 200, and 400 mg/kg	Target NTF	↓: NF-κB/NLRP3,TNF-α, IL-6, IL-1β	Fluoxetine	Yi et al. (2021)
						↑: BDNF/TrkB/ERK/CREB		

Abbreviation: PSP, *Polygonatum sibiricum* polysaccharides; YLSP, *Yulangsan* polysaccharides; ASP, *Angelica sinensis* polysaccharides; SP, sulfated pachymaran; PCAP, *Poria cocos acidic* polysaccharides; LNT, Lentinan; GLP, *Ganoderma lucidum* polysaccharides; LBP, *Lycium barbarum* polysaccharides; LLP, *Lily* polysaccharides; APS, *Astragalus* polysaccharides; DOP, *Dendrobium officinale* polysaccharides; GEP, *Gastrodia elata* polysaccharides; MCP, *Momordica charantia* polysacDOPcharides; OP, Okra polysaccharides; LJP, *Lonicera japonica* polysaccharides; APSP, *Acanthopanax senticosus* polysaccharides; GBP, *Ginkgo biloba* polysaccharides; TG, Total glycosides; DPR, degraded porphyrin.

### 2.1 Polysaccharides regulate the expression of neurotransmitters and their receptors

The mechanism of action underlying the majority of current antidepressants is predicated upon the hypothesis pertaining to the monoaminergic system. According to the hypothesis of the monoaminergic system, depression is characterized by a reduction in levels of 5-hydroxytryptamine, dopamine (DA), and norepinephrine (NE) within the central nervous system. The pathogenesis of depression is rooted in the dysregulation of 5-HT, DA, and NE neurotransmitter systems. The upregulation of type A monoamine oxidase (MAOA) and the downregulation of serotonin (5-hydroxytryptamine, 5-HT) and NE levels in the brain are considered to be the primary etiological factors underlying depression. The MAOA enzyme is responsible for the breakdown of monoamine neurotransmitters, including 5-HT, NE, and DA. It significantly contributes to the pathogenesis, progression, and treatment of various neuropsychiatric conditions. Studies have demonstrated that DA, 5-HT, and other neurotransmitters are crucial in maintaining chemical homeostasis within the brain. An imbalance of these substances, whether it be an excess or deficiency of neurotransmitters, can result in abnormalities within the signaling system of the brain, ultimately leading to the onset and development of depression (Kim et al., 2019).

Serotonin, also known as 5-HT, is a monoamine neurotransmitter that accumulates in nerve endings through the action of the 5-hydroxytryptamine transporter (SERT). This transporter facilitates the uptake of 5-HT into the cytoplasm to replenish synaptic vesicles and terminate its extracellular effects. In addition to regulating behavioral, emotional, and memory processes in the human body, 5-HT also plays a crucial role in the treatment of various psychiatric and neurological disorders and is pivotal in understanding depression’s pathogenesis and treatment. In addition, 5-HT exhibits a distinctive form of neuroplasticity that encompasses synaptic plasticity. It has been demonstrated that addressing the deficit in synaptic plasticity caused by neuronal atrophy and cell death can be beneficial in the treatment of depression (Popa et al., 2010). The substance NE is derived from adrenaline by removing the N-methyl group, and it plays a crucial role in regulating the functions of various internal organs, glands, and the immune system. Additionally, NE serves as a vital neurotransmitter within the central nervous system (CNS). NE neurons originate in the locus coeruleus (LC) of the brain and extend their axons through different regions such as the CNS cortex, hypothalamus, amygdala, cerebellum, and spinal cord to reach primary nerve centers or other neurons (Jovanovic et al., 2022). The serotonin and noradrenaline reuptake inhibitor (SNRI) functions as a modulator of NE neurotransmission, making it an effective first-line medication for depression treatment. Its antidepressant effect is attributed to its regulation of NE levels in the body. DA serves as the predominant catecholamine neurotransmitter in the brain and plays a crucial role in transmitting feelings of excitement and happiness, as well as being involved in learning and rewarding activities. It is closely associated with human eroticism and sensation, transmitting pleasure and excitement while participating in learning, motor function, and reward-related processes. Dopaminergic activity is regulated by the ventral inferior colliculus of the hippocampus and basolateral amygdala regions. Studies have demonstrated deficits within the dopaminergic system among patients with depression, suggesting that these deficiencies may originate from dysfunctions within their afferent circuits (Belujon and Grace, 2017). The existing body of evidence strongly indicates an association between dysfunction in the dopaminergic system and depression. The excitatory neurotransmitter glutamate (Glu), which is found in the central nervous system (CNS), functions as an amino acid neurotransmitter and interacts with both ionotropic and metabotropic receptors. The production of Glu in neurons occurs through glucose-derived intermediates of the tricarboxylic acid cycle and branched-chain amino acids. It is subsequently released at synapses in the brain, exerting short-term effects on postsynaptic excitability and longer-term effects on synaptic strength and neural plasticity. This process involves modulation of the second-messenger system, downstream effects on the activity of various membrane-bound receptors, nuclear gene expression, and translation. Abnormalities in the transmission of excitatory or inhibitory neurotransmitters and neuronal plasticity can result in dysfunctions in brain function, while altered levels of Glu and γ-aminobutyric acid (GABA) have been associated with dysfunction of neural networks. Clinical studies conducted on depression have also identified changes in the concentration and activity of Glu and GABA (Duman et al., 2019), suggesting that dysfunctions in excitatory and inhibitory neurotransmitter signaling mechanisms may play a significant role in depression. The findings of other studies have demonstrated that Glu neurotransmission plays a crucial role in the pathophysiology and therapeutic response to depression, leading to a reduction in depressive symptoms (Fond et al., 2014; Newport et al., 2015). The inhibitory neurotransmitter GABA is naturally present in the human nervous system, acting as a non-protein amino acid responsible for precise control and regulation of excitatory transmission. The neurotransmitter GABA is a significant target for 5-HT afferent fibers (Möhler, 2012), and its physiological effects encompass the modulation of synaptic transmission, facilitation of neuronal development, as well as prevention of insomnia and depression.

Currently, the primary focus of clinical depression treatment lies in targeting monoamine neurotransmitters. This is achieved by inhibiting monoamine oxidation and blocking the reuptake of 5-HT and NE, thereby increasing extracellular levels of 5-HT, DA, and NE throughout the brain. Consequently, there is an elevation in synaptic concentrations of these neurotransmitters which enhances excitatory potential transmission at neural synaptic endings to ameliorate depressive symptoms (Yang et al., 2021).

The *Polygonatum sibiricum* is a traditional medicinal and food plant, wherein the *Polygonatum sibiricum* polysaccharide (PSP) serves as one of its primary bioactive constituents. In the acute behavioral despair mouse depression model, PSP (100, 200, and 400 mg/kg) significantly reduced immobility time in tail-hanging and forced-swimming experiments. Moreover, the levels of 5-HT, DA, and NE in the cortex of mice were significantly elevated compared to those in the model group (Wei et al., 2022). In the lipopolysaccharide (LPS) depression mouse model and the chronic unpredictable stress (CUS)-induced depression mouse model, PSP were found to enhance depressive-like behavior in mice and significantly elevate hippocampal 5-HT levels (Shen et al., 2021; Shen et al., 2022). The aforementioned studies suggest that the antidepressant effects of PSP are associated with the modulation of monoamine neurotransmitters in the brain.

The *Yulangsan* polysaccharide (YLSP, 300 and 600 mg/kg) can also exert antidepressant effects by elevating the levels of 5-HT, DA, and NE in brain tissue (Liang et al., 2010). The *Angelica sinensis* polysaccharide (ASP), one of the primary active compounds derived from Angelica sinensis (Hou et al., 2021), has been found to significantly reduce the duration of forced swimming and immobility in mice with hanging tails at a dosage of 40 mg/kg (Ding et al., 2021). Moreover, it enhances sugar-water preference, increases the levels of DA and 5-HT in the hippocampus, upregulates tryptophan hydroxylase mRNA expression, and elevates the GABA/Glu ratio-an essential rate-limiting enzyme involved in 5-HT synthesis, thereby regulating monoamine neurotransmitter transmission and rectifying excitatory-inhibitory imbalances in mice. The rat depression model was established by (Zhang et al., 2019) through ovarian removal combined with CUS, aiming to explore the correlation between the antidepressant effect of sulfated pachymaran (SP) and AMPA receptors (Zhang et al., 2019). The AMPA receptor, a type of Glu receptor primarily responsible for excitatory synaptic transmission in the brain, is closely associated with the pathogenesis of depression (Shirayama et al., 2022). The administration of SP (50 and 100 mg/kg) for 21 days was found to reduce hippocampal neuronal damage and increase the expression levels of both AMPA receptor Glu R1 and p-Glu R1. However, these anti-inflammatory effects and upregulation of AMPA receptors were completely inhibited by the AMPA receptor inhibitor GYKI52466. These findings suggest that the antidepressant effect of SP may be achieved through the regulation of AMPA receptor Glu R1 expression. *Ganoderma lucidum* polysaccharide (GLP, at doses of 1, 5, and 12.5 mg/kg) also increased the expression levels of p-Glu A1, Glu A1, and Glu A2 in the hippocampus of mice subjected to chronic social frustration stress (Li et al., 2021).

The N-methyl-D-aspartate receptor (NMDAR) is a specific receptor for glutamate, with NR2B being the most important functional subunit. Excessive stress can lead to increased glutamate release and overactivation of NMDAR, resulting in calcium influx and subsequent activation of intracellular signaling pathways that cause neuronal atrophy and death, ultimately leading to mood disorders and depression-like symptoms (Abdoulaye et al., 2021). The administration of *Lycium barbarum* polysaccharide (LBP, 25, 50, and 100 mg/kg) to rats for three consecutive weeks intragastrically was found to effectively inhibit the excessive activation of NR2B in the prefrontal cortex of rats, reduce the expression of calmodulin kinase II (CaMKII), a crucial downstream signaling molecule of NMDAR, and alleviate depressive-like behaviors in rats with PTSD (Chu, 2019).

**FIGURE 2:**
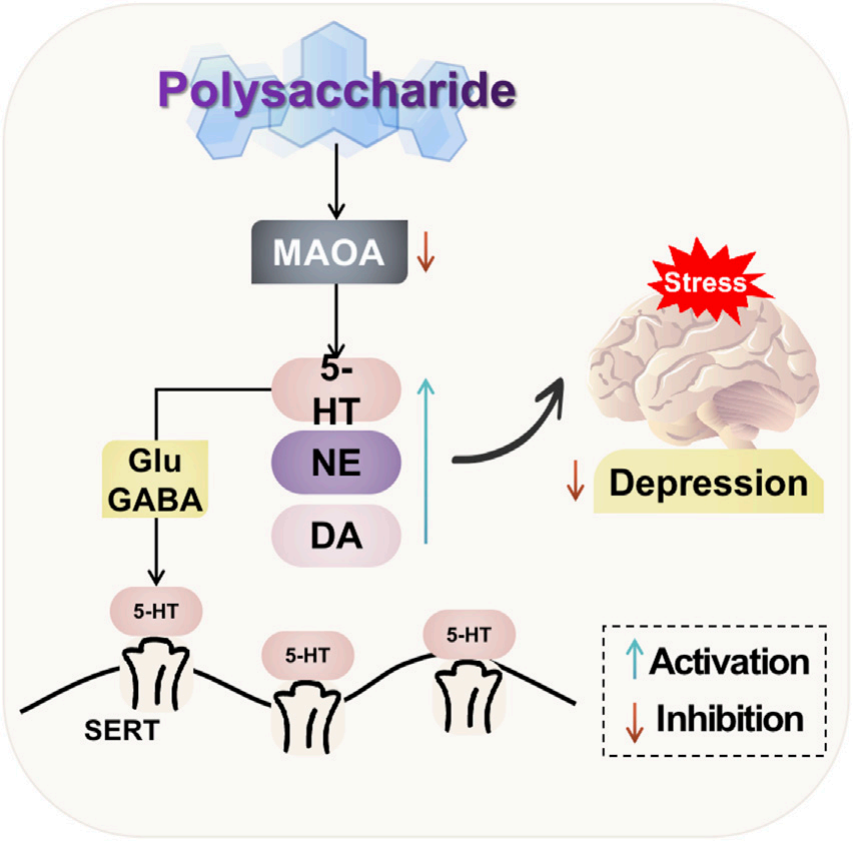
Possible antidepressant mechanism of polysaccharide targeted intervention in neurotransmitters and their receptors.

The natural plant polysaccharides have the ability to modulate neurotransmitters in the brain. They can significantly enhance the levels of 5-HT, DA, and NE; mitigate hippocampal neuronal damage and regulate excitatory synaptic transmission in the brain; as well as exert antidepressant effects. Additionally, natural plant polysaccharides can inhibit excessive activation of NR2B in the prefrontal cortex of rats, reduce expression of the pivotal signaling molecule CaMKII downstream of NMDAR, and ameliorate depressive behaviors induced by post-traumatic stress disorder. The potential mechanism of polysaccharide-targeted intervention in depression through neurotransmitters and their receptors is illustrated in Figure 2.

### 2.2 Polysaccharides regulate the HPA axis

The hypothalamic-pituitary-adrenal (HPA) axis plays a crucial role in stress regulation and serves as a vital component of the neuroendocrine system, which is intricately associated with stress and stimulation (Van Den Eede and Claes, 2004; Mikulska et al., 2021). During prolonged stress and the stress response, corticosterone (CORT) levels remain elevated due to the loss of negative feedback regulation on corticotropin-releasing hormone (CRH) and adrenocorticotropic hormone (ACTH) (Song et al., 2021). This leads to hyperactivity of the HPA axis, increased release of CRH, stimulation of the pituitary gland to release ACTH, and disruption in the normal synthesis, release, and degradation of corticosterone by the adrenal cortex. Consequently, this imbalance results in damage to hippocampal neurons which can trigger or exacerbate depression. In a negative feedback loop, the HPA axis suppresses the feedback signals transmitted by cortisol to the hypothalamus and pituitary gland in order to decrease CRH and ACTH production, thereby regulating its own secretion level for stress response management (Chai et al., 2022). Therefore, implementing strategies to attenuate the release of CORT, CRH, and ACTH while promoting negative feedback regulation of the HPA axis represents efficacious approaches for decelerating the progression of depression.

The administration of Polygonatum sibiricum polysaccharide (PSP) significantly decreased CORT levels in LPS and chronic unpredictable mild stress (CUMS)-induced mice, thereby demonstrating the inhibition of hyperactivity in the HPA axis (Shen et al., 2021). The Lycium barbarum polysaccharide (LBP) significantly downregulated the expression of N-methyl-D-aspartate receptor 2B subunit (NR2B) and calmodulin kinase II (CaMKII) proteins, reduced serum CORT levels, and enhanced the negative feedback regulation of the HPA axis, thereby ameliorating depressive behaviors in rats with post-traumatic stress disorder (PTSD) (Chu, 2019). Liu et al. (2022) discovered that the administration of Lily polysaccharides (LLP), Astragalus polysaccharides (APS), and their combination to CUMS-induced mice for consecutive 28 d significantly ameliorated the depression-like behavior of CUMS mice. The polysaccharides mitigated the pathological damage of neuronal cells in the hippocampal CA1 region to varying extents, and markedly reduced plasma levels of ACTH and CORT. The combined administration of LLP and APS exhibited a superior antidepressant effect compared to the individual administration of polysaccharides. The study conducted by Zhang et al. demonstrated that Dendrobium officinale polysaccharide (DOP) effectively reduced the elevated serum levels of CRH, ACTH, and CORT in depression model mice (Zhang et al., 2022). This suggests that DOP restores the HPA axis and mitigates the depressive effects induced by de-virginized and chronic mild stress-induced peri-menopausal syndrome model mice.

**FIGURE 3:**
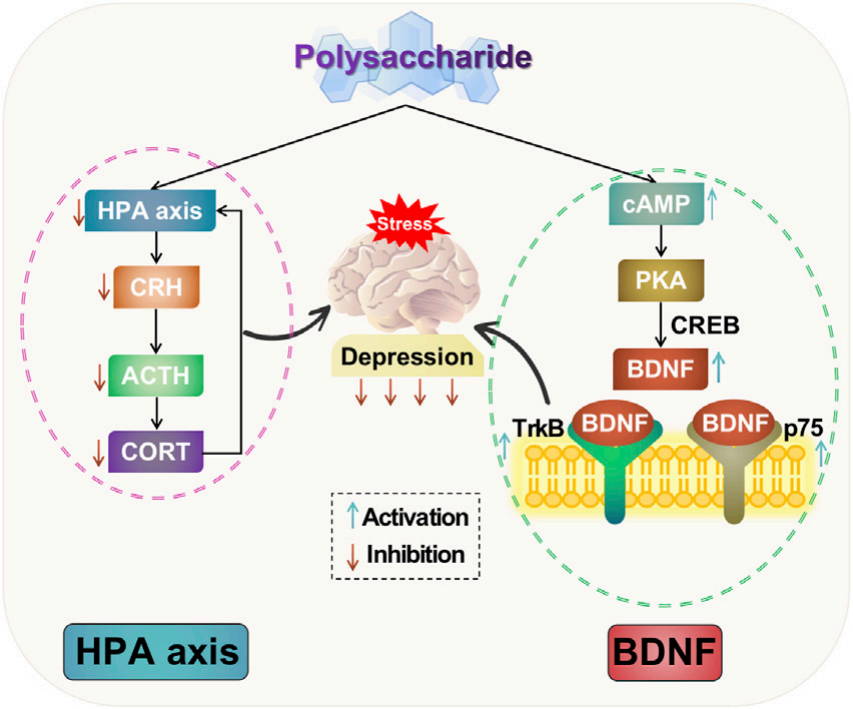
Possible antidepressant mechanism of polysaccharide targeted intervention in HPA axis and neurotrophic factors.

In summary, polysaccharides can ameliorate depressive-like behavior by inhibiting hyperactivity of the HPA axis. They have the ability to decrease levels of CRH, ACTH, and CORT, thereby facilitating negative feedback regulation of the HPA axis. Additionally, polysaccharides can mitigate hippocampal neuronal damage, suppress excessive activation of the HPA axis, exerting antidepressant effects. The potential mechanism of polysaccharide-targeted intervention in depression through HPA axis is illustrated in Figure 3.

### 2.3 Polysaccharides regulate the neurotrophic factors

Neurotrophic factors (NTF), also referred to as neurotrophic agents, are a class of substances that exert a supportive influence on neuronal survival and facilitate neuronal regeneration and functional recovery. They are widely employed in the clinical management of Alzheimer’s disease, cerebral atrophy, Parkinson’s disease, and other neurological disorders (Castrén et al., 2007; PehliVan Karakas et al., 2020). Brain-derived neurotrophic factors (BDNF), the most widely distributed neurotrophic factors in the CNS, plays important roles by activating both the prokinetic myosin-associated kinase (Trk) and p75 receptor. BDNF has become a representative factor in depression research. The blood of patients with persistent and recurrent depression, as well as animal models of depression, have been consistently found to exhibit reduced levels of NTF in numerous studies (Zhao et al., 2021; Zhuang et al., 2022; Joo et al., 2023). The conventional and rapid antidepressants not only rely on the expression of BDNF and its downstream signaling for their efficacy, but they also directly interact with the transmembrane structural domain of the TrkB dimer, leading to the stabilization of a multiprotein complex conformation that facilitates TrkB binding to BDNF. NTF can activate cell signaling pathways through Trk receptors, which in turn regulate various aspects of neuronal function such as cell fate determination, axon growth, dendritic growth and pruning (Dwivedi, 2009).

Neuroplasticity is modulated in individuals with depression, where the ratio of BDNF to pro-BDNF plays a essential role in synaptic plasticity (Vigna et al., 2019). The cyclic AMP (cAMP)/cAMP-response element binding protein (CREB)/BDNF pathway serves as an important antidepressant pathway, as well as a significant route and target for pharmacotherapy in depression. The cAMP/CREB/BDNF is a crucial signaling pathway in regulating hippocampal neuronal regeneration in depression (Yamada et al., 2003). The main mechanism of action of antidepressants is to enhance the concentration of cAMP, thereby inducing alterations in the cAMP signaling cascade and its downstream target, pCREB BDNF (Gao et al., 2022).

The antidepressant effects of *Yulangsan* polysaccharide (YLSP) have been demonstrated in animal models of “behavioral despair” (Liang et al., 2010), exerting a suppressive effect on depression through the upregulation of monoamine neurotransmitters, enhancement of prefrontal cortical adenylate cyclase activity, and increased hippocampal expression of BDNF (Liang et al., 2012). Lu et al. (2013) proposed that the neuroprotective effect of the YLSP group in a chronic stress depression model mice may be attributed to its ability to stimulate the expression of BDNF and its receptor TrkB, thereby activating neuronal protective signaling pathways and CREB activation, which inhibits neuronal death while promoting differentiation and regeneration. The acidic polysaccharides derived from *Poria cocos* were found to enhance the levels of BNDF, 5-HT, 5-HIAA, DA, and NE in the hippocampus while significantly reducing Glu levels. This suggests that these polysaccharides may exert antidepressant effects by modulating relevant trophic factors and neurotransmitters in depressed rats (Chen et al., 2021). Additionally, *Ganoderma lucidum* polysaccharides (GLP) were observed to elevate BDNF expression in the hippocampus of mice subjected to chronic social frustration stress (Li et al., 2021).

The bioactive polysaccharide degraded porphyrin (DPR), extracted from *Porphyra haitanensis*, was utilized by Yi et al. to reverse depressive-like behaviors in LPS-treated mice. This treatment activated the BDNF/TrkB/ERK/CREB signaling pathway in the hippocampus of CUMS mice, offering a potential therapeutic approach for depression (Yi et al., 2021). Yang et al. (2022) discovered that the alcohol-soluble polysaccharides present in *Dendrobium officinale* flowers exhibit additional protective effects against neuronal apoptosis and contribute to the maintenance of the 5-HT system by activating the BDNF/TrkB/CREB pathway. Liu et al. investigated the combined effects of *Lily* polysaccharide (LLP) and *Astragalus* polysaccharide (APS) in a specific ratio on depressive-like behaviors and their modulation of the adenylyl cyclase/cyclic adenosine monophosphate/protein kinase A (AC/cAMP/PKA) signaling pathway in mice subjected to chronic stress (Liu et al., 2022). The findings suggest a significant enhancement in the antidepressant effect of LLP and APS following their combination, potentially through the modulation of brain 5-HT levels and suppression of HPA axis-induced stress, leading to activation of the AC/cAMP/PKA signaling pathway and upregulation of BDNF levels.

The studies suggest that depression induces neuronal atrophy and loss in limbic regions of the brain, such as the hippocampus, prefrontal lobe, and amygdala, along with a decrease in BDNF expression. Conversely, the administration of antidepressant drugs promotes adult hippocampal neurogenesis and an upregulation of BDNF expression. BDNF and relevant signaling pathways may exert antidepressant effects by modulating neuronal growth, differentiation, injury response, apoptosis, and regulating neuroendocrine networks. Therefore, investigating BDNF and relevant signaling pathway is crucial for studying the pathogenesis of depression. The potential mechanism of polysaccharide-targeted intervention in depression through neurotrophic factors is illustrated in Figure 3.

### 2.4 Polysaccharides regulate the neuroinflammatory

Neuroinflammation is an innate immune response of the nervous system that plays a pivotal role in the pathogenesis of numerous neuropsychiatric disorders (Troubat et al., 2021). Through extensive research on depression, it has been discovered that dysregulated secretion of inflammatory factors can contribute to its onset. The neuroinflammatory response assumes a critical role in mediating depression, as evidenced by elevated levels of pro-inflammatory cytokines within the central nervous system and aberrant activation of astrocytes and microglia (Benedetti et al., 2020). These findings suggest that neuroinflammation can either induce intracerebral lesions or be modulated for repairing intracerebral injuries.

Microglia are specialized cells of the central nervous system that exhibit macrophage-like properties, playing a crucial role in neuroinflammation. In response to stress, infection, trauma, or injury, microglia undergo phenotypic changes known as “microglial activation” and “microglial polarization,” which involve their conversion into pro-inflammatory (M1) and anti-inflammatory (M2) states (Orihuela et al., 2016; Feng et al., 2020; Zhou et al., 2020). The M1 phenotype of microglia is responsible for synthesizing and releasing various cytokines, including prostaglandin E2 (PGE2), CRP, and TNF-alpha, into the bloodstream (Attwells et al., 2020). Maintaining a balanced and stable state of M1/M2 microglia is crucial for normal immune function in the central nervous system. Evidence suggests that an increase in M1 microglia is associated with depression (Guo et al., 2020). The CUS activates microglia and induces depressive behaviors (Xiao et al., 2021), leading Yirmiya et al. to propose that depression can be characterized as “microgliosis” (Yirmiya et al., 2015). These studies indicate that the activation of microglia plays a significant role in the pathogenesis of depression.

Astrocytes and microglia play a crucial role in regulating inflammation within the nervous system through the secretion of various cytokines and inflammatory mediators, such as IL-1, TNF-α, and complement component 1q. The transcriptional responses of astrocytes are induced by the secretion of these molecules from microglia. Additionally, this release of inflammatory mediators leads to a decrease in phagocytosis activity and expression of neurotrophic factors (Linnerbauer et al., 2020). Animal experiments have demonstrated that inhibiting astrocyte activation can improve depressive symptoms (Wang et al., 2019b). However, there is currently limited research investigating the mechanisms underlying astrocyte action in neuroinflammation.

The nucleotide-binding oligomerization structural domain-like receptor protein 3 (NLRP3) inflammatory pathway plays a crucial role in the initiation and progression of depression (Bian et al., 2022). Activated NLRP3 inflammasomes cleave pro-IL-1β into active IL-1β, thereby triggering inflammation and concurrently promoting microglial activation while inhibiting hippocampal neurogenesis (Tastan et al., 2021).

Pro-inflammatory factors such as interleukin-1β (IL-1β), interleukin-6 (IL-6), and tumor necrosis factor-alpha (TNF-α) are regarded as depression biomarkers (Carniel and da Rocha, 2021). The excessive production of pro-inflammatory factors leads to neuronal damage, apoptosis, disruption in neurotransmitter transmission, activation of the HPA axis, alteration in related signaling pathways, and exacerbation of various subtypes of depression. These processes primarily involve the phosphatidylinositol-3-kinase/protein kinase B (PI3K/Akt), nuclear factor-kappa B (NF-kB), NLRP3/ASC/caspase-1, and mitogen-activated protein kinases (MAPKs) signaling pathways.

The *Acanthopanax senticosus* polysaccharides (APSP) demonstrated a reduction in the levels of pro-inflammatory factors IL-1β, IL-6, and TNF-α, while simultaneously increasing the expression of p-PI3K, p-Akt, and p-mTOR proteins induced by chronic mild agnostic stress stimuli in rats (Ding et al., 2022). These findings suggest that the antidepressant effects of spikenard polysaccharides are associated with their modulation of the PI3K/Akt/mTOR pathway and anti-inflammatory properties.

*Ganoderma lucidum* polysaccharides (GLP) significantly mitigated the downregulation of pro-inflammatory cytokines in BV-2 microglia induced by LPS or aβ, while promoting the expression of anti-inflammatory cytokines in both BV-2 and primary microglia (Cai et al., 2017). Furthermore, GLP attenuated inflammation-associated microglial migration, morphological alterations, and phagocytosis potential. Studies have demonstrated that GLP possesses a modulatory effect on LPS and aβ-induced neuroinflammation, suggesting its potential antidepressant effects through modulation of microglial inflammatory and behavioral responses to achieve neuroprotective functions. Other studies have confirmed that GLP possess the ability to alleviate depressive symptoms in mouse models, inhibit microglial activation, and promote astrocyte proliferation (Li et al., 2021). Additionally, it downregulates the expression of pro-inflammatory cytokines IL-1β and TNF-α in the hippocampus of mice while up-regulating the expression of anti-infective cytokines IL-10 and BDNF.

The expression levels of AMPA receptor GluR1 and p-GluR1 were significantly elevated by sulfated pachymaran (SP), whereas the anti-inflammatory and up-regulatory effects of SP on AMPA receptors were completely inhibited by the AMPA receptor inhibitor GYKI 52466 (Zhou and Li, 2020). These findings suggest that the antidepressant effect of SP may be achieved through its anti-inflammatory properties and regulation of AMPA receptor GluR1 expression. *Poria cocos* acidic polysaccharides (PCAP) significantly reduced the mRNA and protein expression levels of NLRP3, ASC, caspase-1, IL-1β, and IL-18 in the prefrontal cortex of rats in a chronic unpredictable stress model (Chen et al., 2021). Conversely, they significantly increased the mRNA and protein expression levels of pro-caspase-1, pro-IL-1β, and pro-IL-18. These results indicate that PCAP may inhibit the NLRP3 inflammasome pathway and reverse serum levels of inflammatory factors by regulating associated mRNA and protein expression levels related to NLRP3.

*Gastrodia elata* polysaccharides (GEP) exhibited the ability to attenuate the relative mRNA expression of pro-inflammatory cytokines TNF-α and IL-1β in hippocampal tissues of depressed mice, thereby exerting a neuroprotective effect and ameliorating LPS-induced depressive-like behaviors in mice (Liu et al., 2021). *Momordica charantia* polysaccharides (MCP, 200 and 400 mg/kg) also decreased hippocampal levels of IL-1β, IL-6, and TNF-α, alleviating depressive behaviors in mice subjected to chronic social frustration stress (Deng et al., 2019). Additionally, MCP significantly upregulated PI3K activity and Akt phosphorylation in the hippocampus of depressed mice with chronic social frustration stress. Notably, partial inhibition of the antidepressant effects of MCP was observed upon treatment with LY294002, a PI3K inhibitor.

The intervention of *Astragalus* polysaccharide (APS) significantly reduced the levels of hippocampal TNF-α, IL-1β, and IL-6 in rats with CUS-induced depression (Wang et al., 2018; Wang et al., 2019a; Liu et al., 2019). Additionally, it also significantly decreased the levels of NF-κB p65, p-NF-κB p65, p-IκBα, and the DNA-binding activity of NF-κB p65. Notably, p65 is a subunit of NF-κB that plays a crucial role in this process. These findings suggest that the antidepressant effect of APS may be attributed to their ability to inhibit overactivation of NF-κB signaling pathway as well as regulate downstream inflammatory factors. APS intervention also effectively reduced the expression levels of hippocampal ERK1/2, JNK, and p38 along with their phosphorylated forms in LPS-induced depressed rats (Wang et al., 2018). Moreover, it significantly decreased the levels of downstream phosphorylated c-Fos and c-Jun compared to the model group. This indicates that APS can further suppress Activator Protein 1 (AP-1) by inhibiting MAPK signaling pathway to modulate inflammatory responses and thereby ameliorate depressive-like behaviors.

*Polygonatum sibiricum* polysaccharide (PSP) may exert antidepressant effects by inhibiting the activation of NF-κB expression and nuclear translocation in mouse models of depression induced by LPS and CUS (Shen et al., 2021; Shen et al., 2022). Additionally, PSP decreases the expression levels of pro-inflammatory factors IL-1β and TNF-α in hippocampal tissues. Endogenous ligands produced during brain injury activate Toll-like receptor 4 (TLR4), which in turn activates NF-κB through the MyD88-dependent signaling pathway, leading to the transcription of numerous pro-inflammatory factors (Brancato et al., 2013). PSP demonstrated inhibitory effects on the upregulation of NLRP3, ASC, caspase-1, cleaved-caspase-1, and IL-1β in LPS-induced depression model in mice (Shen et al., 2022). Additionally, they downregulated the expression of Iba-1 as a microglial activation marker and GFAP as an astrocyte activation marker, thereby suppressing the activation of microglia and astrocytes. Simultaneously, PSP exhibited potential to ameliorate depressive-like behavior by inhibiting ERK phosphorylation-mediated NF-κB activation and modulating inflammatory responses (Shen et al., 2021).

Yan et al. demonstrated that intervention with Okra polysaccharides (OP) from *Abelmoschus esculentus* (L.) Moecnch suppressed the expression of TLR4, MyD88, and nuclear translocation of NF-κB, suggesting that the TLR4/NF-κB pathway may be involved in the mechanism of action of OP in the brain (Yan et al., 2020). Furthermore, it has been shown that OP can significantly reduce inflammatory factor levels in mice with chronic unpredictable stress-induced depression and concurrently downregulate phosphorylated expression of ERK1/2, JNK, and p38. This suggests that OP may exert their antidepressant effects by modulating inflammatory responses through the MAPKs pathway.

Liu et al. demonstrated that *Lonicera japonica* polysaccharides (LJP) significantly ameliorated depression-like behavior in CUMS model mice (Liu et al., 2019). Furthermore, LJP upregulated the number of hippocampal metameres and protected their structure and arrangement from disruption. There was a significant reduction in the expression of proteins such as NLRP3, Caspase-1, and IL-1β upon treatment with LJP. These findings suggest that LJP may exert an antidepressant effect by inhibiting the NLRP3 inflammatory vesicle-mediated immune-inflammatory response.

**FIGURE 4:**
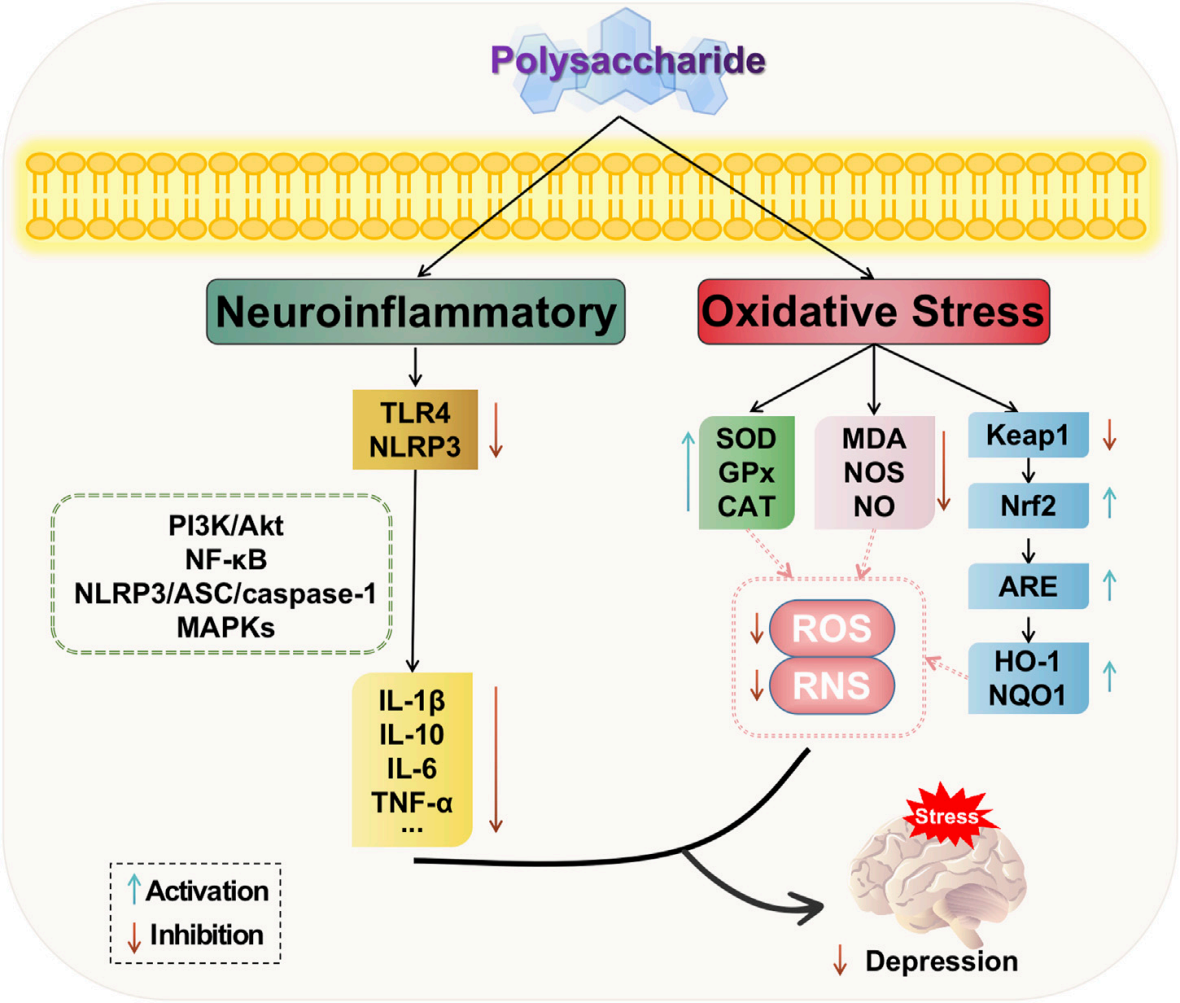
Possible antidepressant mechanism of polysaccharide targeted intervention in neuroinflammatory and oxidative stress.

In summary, natural plant polysaccharides can modulate the PI3K/AKT and NF-κB signaling pathways, thereby reducing the release of inflammatory factors and decreasing the downstream expression levels of inflammation-related proteins. Additionally, polysaccharides can attenuate the phosphorylated expression of ERK1/2, JNK, and p38 in the MAPKs family, thus influencing neuroinflammatory responses. Moreover, they can inhibit protein expression related to the NLRP3/ASC/caspase-1 signaling pathway, consequently impacting immune inflammation and exerting antidepressant effects. Therefore, polysaccharides play a crucial role in regulating inflammation and their ability to suppress inflammatory responses is an integral part of depression treatment. The potential mechanism of polysaccharide-targeted intervention in depression through neuroinflammatory is illustrated in Figure 4.

### 2.5 Polysaccharides regulate the oxidative stress

The “oxidative stress hypothesis of depression” posits that oxidative stress is accountable for the structural alterations in the brains of individuals with depression. Oxidative stress refers to an imbalance between the oxidizing and antioxidant effects within the body, leading to tissue damage. Elevated levels of oxidative stress result in an excess production of reactive oxygen radicals, known as reactive oxygen species (ROS), and reactive nitrogen radicals, referred to as reactive nitrogen species (RNS), which have potential neurotoxicity (Bhatt et al., 2020). The role of oxidative stress is pivotal in the pathological changes associated with stress-related diseases, and it constitutes a significant component in the pathogenesis of depression (Kim et al., 2016). Clinical studies investigating oxidative stress in depression have demonstrated that a key characteristic of oxidative stress in depression is the diminished antioxidant capacity and inadequate blood levels of antioxidant enzymes, such as superoxide dismutase (SOD), glutathione peroxidase (GPx), and catalase (CAT). Conversely, serum levels of lipid markers associated with oxidative stress are elevated, including reactive oxygen species (ROS) and nitrogen species (NS), such as malondialdehyde (MDA), Malondialdehyde (MDA), nitric oxide (NO) and nitric oxide synthase (NOS) (Moylan et al., 2014; Nobis et al., 2020; Manosso et al., 2022). Additionally, there is evidence of protein, DNA, and mitochondrial damage; along with secondary autoimmune responses targeting redox-modified nitrosylated proteins and oxidation-specific epitopes. Therefore, the reduction of oxidative stress damage through elevation of antioxidant levels such as SOD and GSH, along with inhibition of MDA production, contributes to alleviating depressive symptoms in PSD. Moreover, nuclear factor erythroid 2-related factor 2 (Nrf2), a transcription factor, emerges as a pivotal regulator of antioxidant signaling pathways and holds promise as a therapeutic target for depression (Bouvier et al., 2017; Zuo et al., 2022; Luo et al., 2023).

It has been observed that conventional pharmaceutical drugs exhibit limited efficacy in alleviating symptoms and may even induce severe adverse reactions in 30%–60% of patients (Liang et al., 2021). Medicinal plants and their bioactive constituents offer substantial health-promoting advantages, thereby warranting their utilization for the management of various ailments such as cancer, gastrointestinal disorders, and hepatic injury (Cheema and Singh, 2021; Khosravifarsani et al., 2022; Parganiha et al., 2022). Plant-derived polysaccharides represent crucial active components possessing anti-inflammatory, antioxidant, and immunomodulatory properties (Farahani et al., 2015; Wang et al., 2022a; Liaqat et al., 2022b), with notable evidence supporting their potential antidepressant effects.

In recent years, *Polygonatum sibiricum* has been utilized in various formulations for the treatment of depression with promising outcomes (Liu et al., 2022). It encompasses multiple bioactive constituents such as polysaccharides, steroidal saponins, and flavonoids. *Polygonatum sibiricum* polysaccharide (PSP) is a key constituent of PS and exhibits diverse biological activities including antitumor, antioxidant, anti-inflammatory, immunomodulatory effects, as well as regulation of blood glucose and lipids. Shen et al. discovered that the administration of PSP effectively reversed the alterations in reduced SOD levels and increased MDA levels induced by LPS-induced depression model in rats (Shen et al., 2021). PSP may exert its preventive effects on depressive-like behavior through the inhibition of reactive oxygen species (ROS), hyperfunctioning HPA axis, and ERK/NF-κB-mediated inflammatory response. Additionally, Shen et al. employed the LPS and CUMS-induced model to investigate the antidepressant effects of PSP and elucidate its underlying mechanism of action. Their findings revealed that PSP exerts its antidepressant properties by modulating the oxidative stress-calpain-1-NLRP3 signaling axis (Shen et al., 2022). These studies provide experimental foundation for the development of efficacious antidepressant medications.

The *Lycium barbarum* polysaccharide (LBP) is a bioactive compound derived from the *Lycium barbarum* L., which is widely used in traditional Chinese medicine. It possesses various pharmacological properties, including immunomodulatory and anti-aging effects (Xiao et al., 2022). Additionally, LBP exhibits neuroprotective properties that can be attributed to its antioxidant and anti-inflammatory activities (Zhu et al., 2015; Zhao et al., 2017). For instance, studies have demonstrated the protective effects of LBP in models of partial optic dissection injury and focal cerebral ischemic injury (Chu et al., 2013). Recent studies have indicated that LBP may possess therapeutic potential in the treatment of depression (Fu et al., 2021; Li et al., 2022). Zhao et al. conducted an assessment on the antidepressant activity of LBP in rifampicin-induced depressed mice, and proposed a possible mechanism of action whereby LBP attenuates the reduction in apoptosis inhibitors Bcl-2 and PARP by suppressing lipid peroxidation (LPO) production, subsequently leading to a decrease in apoptosis within striatal neurons (Zhao et al., 2019).

The authors hypothesized that *Astragalus* polysaccharide (APS) may exert protective and antidepressant effects on the hippocampus by inhibiting CMUS-induced oxidative stress, which is achieved through APS activation of the Nrf2-ARE pathway in depressed rats (Su et al., 2021). After the study, it was observed that APS intervention significantly upregulated hippocampal Nrf2 gene expression, total Nrf2 protein levels, and nuclear translocation in depressed rats. This finding suggests that APS enhances Nrf2 activation and its subsequent translocation to the nucleus, thereby promoting the antioxidative stress mechanism in rats. Furthermore, APS demonstrated regulatory effects on SOD, GSH-Px, CAT, HO-1 enzymes, indicating its ability to activate the Nrf2-ARE pathway in the hippocampus of depressed rats. Additionally, intervention with *Acanathopanax senticosus* polysaccharides significantly increased CAT and SOD activities while effectively reducing malondialdehyde (MDA) levels in rat hippocampal tissues [69]. The intervention of Lentinan (LNT, 2.5 and 5 mg/kg) also significantly enhanced the activity of superoxide dismutase (SOD) and reduced the levels of malondialdehyde (MDA) in the hippocampus of mice with chronic unpredictable stress-induced depression (Ma et al., 2015).

Based on the aforementioned studies, natural plant polysaccharides exert anti-oxidative stress effects by upregulating the levels of antioxidant enzymes such as superoxide dismutase (SOD), glutathione peroxidase (GSH-Px), and catalase (CAT), while downregulating the levels of malondialdehyde (MDA) as an oxidizing agent. Polysaccharides possess the ability to modulate the oxidative stress response in the depressed hippocampus through multiple pathways, with potential targets including Nrf2-related pathways for polysaccharide-based treatment of depression. The potential mechanism of polysaccharide-targeted intervention in depression through oxidative stress is illustrated in Figure 4.

### 2.6 Polysaccharides regulate the tryptophan metabolism

Tryptophan (TRP) is one of the essential amino acids in the human body and serves as a precursor to 5-HT. Being the sole “raw material” for 5-HT synthesis, it also plays a significant role in the development of depression. Tryptophan undergoes metabolism at three primary sites: the brain, intestine, and liver; among these, the colon stands out as the most crucial site for TRP absorption (O’Mahony et al., 2015; Comai et al., 2020). There are two primary metabolic pathways for tryptophan (Breda et al., 2016; Agus et al., 2018), namely the serotonin pathway and the kynurenine (KNY) pathway (KP). The former involves conversion of tryptophan to 5-HT by tryptophan hydroxylase (TPH), followed by metabolism to 5-hydroxyindoleacetic acid (5-HIAA) via monoamine oxidase in blood. The latter pathway entails generation of kynurenine from tryptophan through the action of indoleamine-2,3-dioxygenase (IDO) or tryptophan-2,3-dioxygenase (TDO). The conversion of tryptophan to kynurenine is catalyzed by the enzyme kynurenine-3-monooxygenase, resulting in the production of 3-hydroxykynurenine (3-HK), which has been implicated in oxidative stress and neurotoxicity. Only a small fraction, less than 5%, of tryptophan is converted to 5-HT, with the majority being metabolized through the KP—the primary route for tryptophan metabolism (Höglund et al., 2019). Studies have demonstrated that acute TRP depletion is associated with the emergence of depressive symptoms, potentially attributed to a significant reduction in 5-HT production (Chen et al., 2022). Simultaneously, an elevation in KYN, another metabolite of TRP, contributes to the development of depressive symptoms through immune dysregulation and induction of neuroinflammation (Schwarcz et al., 2012; Gong et al., 2023). It is evident that there exists a strong correlation between TRP metabolism and depression.

The regulation of key enzyme activities by natural plant polysaccharides can contribute to the modulation and equilibrium of metabolites with specific neuroactive properties, thereby promoting TRP metabolism to 5-HT while inhibiting TRP metabolism to 3-HK (Liaqat et al., 2022a).

Intervention with *Polygonatum sibiricum* polysaccharides (PSP) was found to downregulate TRP and 3-HK levels in the hippocampus of mice in a behavioral despair model (Wei et al., 2022). Furthermore, considering the concurrent increase in 5-HT levels, it is postulated that these polysaccharides may modulate TRP metabolism towards the TRP/5-HT pathway by enhancing TPH enzyme activity and inhibiting IDO enzyme activity. PSP may attenuate kynurenine metabolism and reduce downstream 3-HK levels, thereby exerting their antidepressant effects. The total glycosides (TG) of *Cistanche tubulosa* significantly elevate serum TRP levels in chronically unpredictable stressed rats (*p* < 0.05), while reducing the levels of serum KYN and KYN/TRP ratio (*p* < 0.05) (Fan et al., 2021). Simultaneously, they downregulate the expression of IDO protein in the colon and hippocampus of rats (*p* < 0.05). These findings suggest that these glycosides possess an inhibitory effect on TRP metabolism into KNY, thereby promoting increased conversion of TRP to serotonin and exerting an antidepressant effect. Chen et al. demonstrated the specific protective effect of Tongxieyaofang polysaccharide in mitigating TRP metabolism disorders induced by CUS (Chen et al., 2023). They observed that the administration of polysaccharide solution effectively suppressed the transcriptional activity of IDO1 in the colon, thereby preventing biased TRP metabolism towards the KP. This led to a reduction in serum KYN concentration and KYN/TRP ratio, ultimately resulting in decreased colonic 5-HT levels, increased hippocampal 5-HT levels, and alleviation of depressive symptoms.

Cheng et al. obtained Tiansi Liquid, formulated with *Morinda officinalis* How polysaccharides and *Cuscuta chinensis* polysaccharides in a 1:1 ratio. After conducting a preliminary investigation, Tiansi liquid exhibits certain effects on monoamine neurotransmission and neuronal morphology in brain tissue by inhibiting the activity of IDO and reducing the expression of its mRNA (Zhou et al., 2015). The activity of IDO is reduced, leading to a decrease in the expression of its mRNA. This results in IDO inhibition, which regulates the TRP/KYN pathway and subsequently impacts monoamine neurotransmitter transmission and neuron morphology in brain tissue, thereby exerting a certain antidepressant effect. Subsequently, the groups investigated the antidepressant mechanism of Tiansi liquid on a hydrocortisone-induced depression rat model (Cheng et al., 2018). The metabolomics results demonstrated that Tiansi liquid effectively downregulated TDO, IDO, and quinoline (QUIN) levels while simultaneously reducing the KYN/TRP ratio. It significantly increased kynurenic acid and 5-HT levels. These findings suggest that Tiansi liquid alleviates depressive symptoms in rats by modulating gut microbiota composition and metabolites within the TRP/KYN pathway.

**FIGURE 5:**
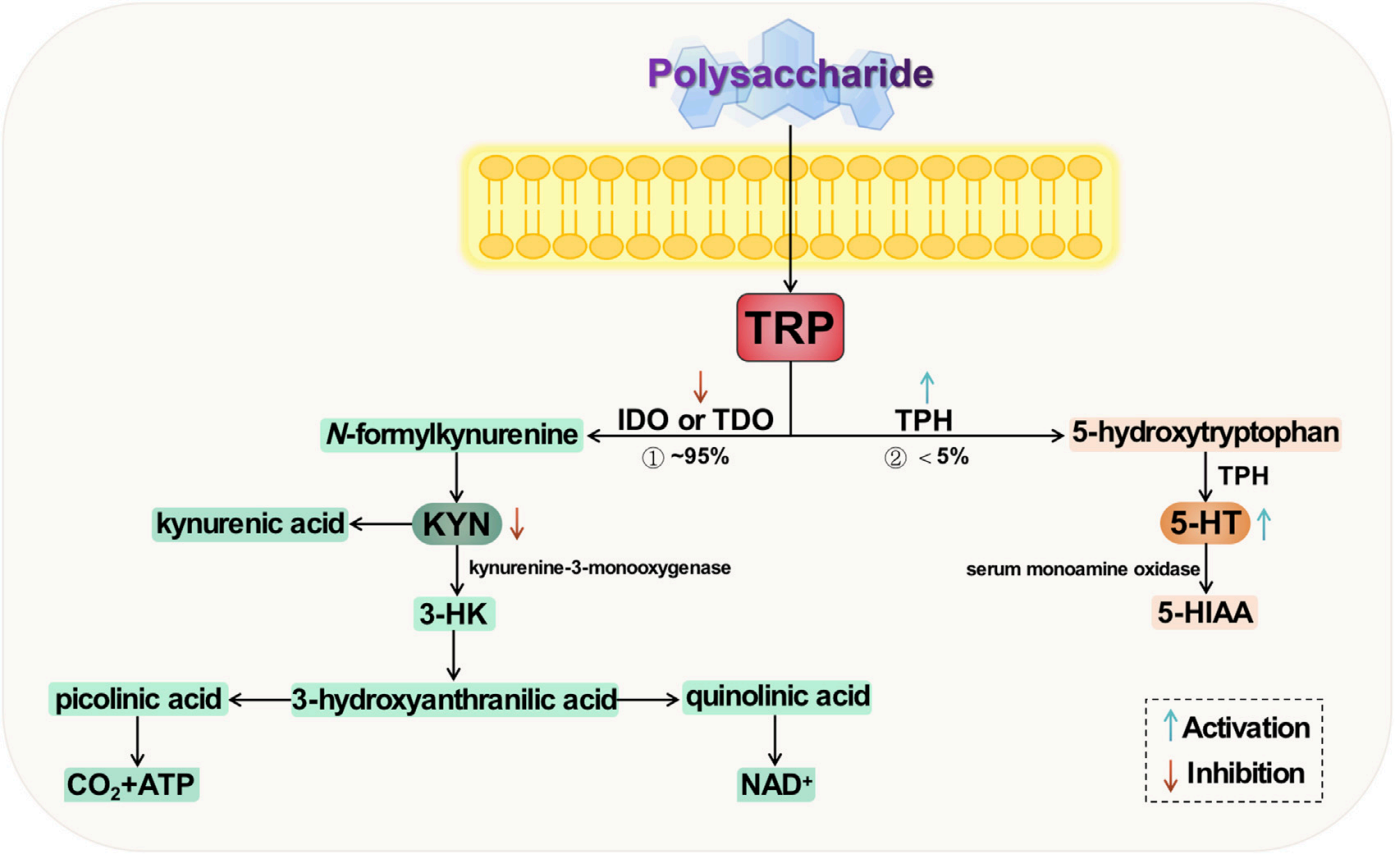
Possible antidepressant mechanism of polysaccharide targeted intervention in tryptophan metabolism.

Tryptophan metabolism is implicated in several hypotheses regarding the pathogenesis of depression. Firstly, under stress conditions, TRP conversion to 5-HT and KYN is affected, leading to decreased expression of TPH and insufficient production of 5-HT, resulting in a deficiency of monoamine transmitters. Secondly, excessive TRP to KYN conversion is closely associated with the body’s inflammatory response and overexpression of IDO. Lastly, downstream products of KYN exert excitotoxic effects that can trigger apoptosis through activation of ionotropic glutamate receptors impacting neuroplasticity. The two key enzymes, TPH and IDO, exert control over the direction of TRP metabolism, which is a dynamic factor in depression and various other neuropsychiatric disorders. Consequently, these enzymes may emerge as potential targets for future research on natural plant polysaccharides intervention in depression. The potential mechanism of polysaccharide-targeted intervention in depression through tryptophan metabolism is illustrated in Figure 5.

### 2.7 Polysaccharides regulate the gut microbiota

The gut serves as a habitat for parasitic or commensal bacteria and plays a crucial role in maintaining the homeostasis of the internal milieu (Ji et al., 2020). The gut establishes a bidirectional regulatory mechanism with the brain through the mediation of gut microbiota, known as the microbiota-gut-brain axis (MGBA). This axis encompasses the central nervous system, enteric nervous system, autonomic nervous system, as well as neuroendocrine, enteroendocrine, and neuroimmune systems (Appleton, 2018). The gut microbiota is influenced by stress, leading to changes in its composition and diversity. Disruption of the gastrointestinal microbiota can activate the gut-brain axis, resulting in alterations in mental mood and depressive behaviors (Pferschy-Wenzig et al., 2022). The modulation of the gut-brain axis (MGBA) involves the integration of neural, hormonal, immune signals, and other factors between the gut and brain. This provides a potential pathway for the entry of gut microbiota and its metabolites into the brain. Consequently, regulating MGBA has emerged as a promising approach for treating psychiatric disorders such as depression (Rathour et al., 2023).

The gut microbiota communicates with the brain by activating the HPA axis, altering neurotransmitters, producing microbial metabolites, influencing neurotrophic factors, modulating immune and inflammatory responses, and engaging vagus nerve pathways to regulate brain function and activity (Han et al., 2022). These interactions subsequently impact the pathogenesis of depression. Dysbiosis of the gut microbiota can lead to hyperactivity of the HPA axis, resulting in elevated levels of ACTH and CORT, as well as reduced BDNF. Conversely, nutritional therapy involving the administration of beneficial bacteria such as Bifidobacteria, *Lactobacillus*, and probiotics can attenuate HPA axis activity, restore normal ACTH, CORT, and BDNF levels, and exert an antidepressant effect (Sun et al., 2022). Dysbiosis of the gut microbiota also leads to dysfunction of specific cellular microbiota (such as enterochromaffin cells and *citrobacter*), thereby impacting the involvement of gut microbiota in the synthesis and secretion of key neurotransmitters and factors, including GABA, Glu, NE, 5-HT, and DA (Rajanala et al., 2021). These dysfunctions contribute to the development of depression. The metabolites produced by the gut microbiota, such as short-chain fatty acids (SCFAs), play a crucial role as signaling molecules that regulate the production of intestinal peptides through enteroendocrine cells (EECs) (Averina et al., 2020). These peptides are responsible for governing the gut-brain axis and stimulating the synthesis of gut-derived 5-HT by enterochromaffin cells (ECs), subsequently influencing hormone communication between the gut and brain (Eicher and Mohajeri, 2022). Thus, a healthy gut microbiota promotes brain development and regulation. Disrupted gut microbiota can impact the expression levels of BNDF, potentially leading to depression (Zhao et al., 2022). Therefore, the treatment of depression may involve regulating the balance of intestinal microbiota, modulating BDNF-related pathways, and promoting both BDNF secretion levels and gene expression. The communication between gut microbiota and the central nervous system (CNS) is facilitated by neuroactive substances produced by the former through the vagus nerve (Tan et al., 2022). Neurotoxic metabolites produced by gut microbiota can be transmitted to the central nervous system (CNS) through the vagus nerve, thereby influencing brain function and inducing depression. Disturbances in gut microbiota may result in the release of bacteria into the bloodstream, leading to excessive production of LPS, which causes oxidative stress and inflammatory responses. These processes can disrupt the blood-brain barrier, allowing inflammatory factors to enter the CNS and subsequently activate neuroglia through various signaling pathways such as NF-κB and cholinergic mechanisms, ultimately promoting the development of depression (Han et al., 2022).

A variety of herbal compounds, monotherapies, and their active ingredients have demonstrated remarkable efficacy in reducing depression symptoms with minimal side effects when utilizing traditional herbal treatments (Bi et al., 2022; Shao et al., 2022). Natural plant polysaccharides can effectively regulate gut microbiota disorders and intervene in the development of depression through MGBA. The intervention of natural plant polysaccharides to improve imbalances in gut microbiota provides a novel target for the prevention and treatment of depression (Sun et al., 2021).

Yan et al. discovered that Okra polysaccharides (OP) from *Abelmoschus esculentus* (L.) Moecnch exhibited significant improvements in depression-like behavior and alterations in the structure and abundance of gut microbiota within a depressed mouse model of CUMS (Yan et al., 2020). Specifically, at the phylum level, there was a notable reduction in the relative abundance of Bacteroidetes and Actinobacteria, accompanied by an increase in the relative abundance of Firmicutes. At the genus level, there was a significant decrease in the relative abundance of Barnesiella and *Bacteroides*, while *Lactobacillus* showed a substantial increase. The relative abundance of *Lactobacillus* was increased, while the levels of Barnesiella and *Bacteroides* were decreased by OP. The antidepressant effects mediated by gut microbiota were further elucidated through fecal microtransplantation technique (FMT). Furthermore, OP significantly reversed the reduction in acetic acid, propionic acid, and butyric acid levels, as well as the elevation in isovaleric acid level within the intestines of CUMS mice. These findings suggest that OP may exert their antidepressant effects by modulating both the composition of gut microbiota and the levels of various metabolites such as SCFAs. Chen et al. discovered that the administration of *Ginkgo biloba* polysaccharides (GBP, 300 mg/kg) for consecutive 28 days effectively mitigated the antidepressant effects induced by chronic stress in mice, while also exerting antidepressant effects through rectifying dysregulated gut homeostasis, elevating brain levels of 5-HT and DA, and enhancing the abundance of *Lactobacillus* in the intestine (Chen et al., 2019). The administration of Total *Cistanche* polysaccharides effectively mitigated the inflammatory response in the colon and ameliorated gut barrier disruption by modulating the dysbiosis of gut microbiota in rats subjected to chronic unpredictable stress, indicating that the antidepressant effect of *Cistanche* polysaccharides is also associated with their regulation on gut microbiota (Fan et al., 2021). The polysaccharides derived from *Polygonum sibiricum* exhibited the ability to suppress depression-like behavior in perimenopausal mice induced by ovariectomy plus chronic mild stress (Zhang et al., 2022). Additionally, they demonstrated the capacity to mitigate the inflammatory response via modulation of the MGBA and inhibit excessive activation of the HPA axis. Zhang and others discovered that *Polygonum sibiricum* polysaccharides (PSP) were capable of elevating serum levels of 5-HT and NE, reducing pro-inflammatory cytokine levels in the hippocampus, as well as inhibiting the PI3K/AKT/TLR4/NF-κB and ERK/CREB/BDNF pathways in mice with depression induced by CUMS (Zhang et al., 2023). The PSP compound exerts antidepressant-like behavior by modulating the MGBA axis.

**FIGURE 6:**
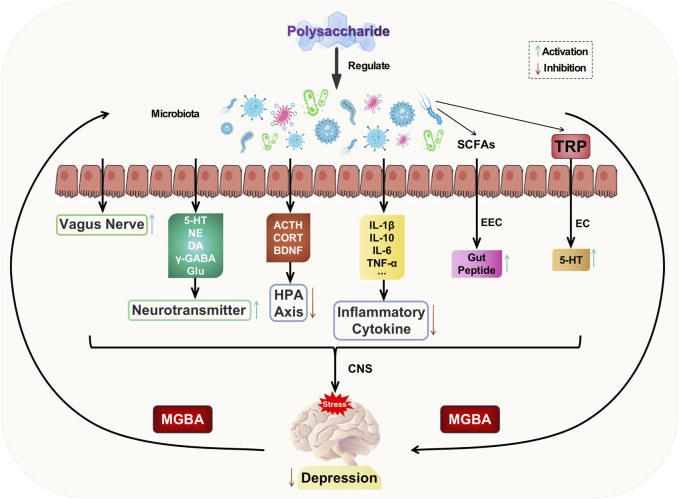
Possible antidepressant mechanism of polysaccharide targeted intervention in gut microbiota.

Based on the aforementioned studies, it can be concluded that natural plant polysaccharides have a significant impact on regulating gut microbiota disorders, improving depressive symptoms through the MGBA axis, and playing a crucial role in modulating host-microbe crosstalk. Current research exploring the relationship between depression and various gut microbiota is still in its preliminary stage. The specific key microbiota responsible for depression has not been identified, leaving ample room for further investigation into utilizing gut microbiota regulation as a broader therapeutic approach to treating depression. Polysaccharide intervention aimed at ameliorating imbalances in gut microbiota offers a novel therapeutic strategy for both preventing and treating depression. The potential mechanism of polysaccharide-targeted intervention in depression through gut microbiota is illustrated in Figure 6.

## 3 Conclusion and prospects

Due to the intricate pathogenesis of depression, the current antidepressants exhibit limited efficacy and are associated with potential toxic side effects. Hence, there is an urgent need to develop drugs that can effectively prevent or treat depression while minimizing adverse reactions. Natural plants derived from diverse sources offer minimal harm to the human body and possess the advantage of being multi-targeted in their actions. Several traditional Chinese medicine polysaccharide drugs, such as *Astragalus* polysaccharide injection, Ginseng polysaccharide injection, and *Poria cocos* acidic polysaccharide oral solution, have been developed for clinical use due to their immunomodulatory effects in improving chemotherapy-induced immune deficiency among tumor patients. The discovery has revealed that natural plant polysaccharides possess the ability to exert antidepressant effects through the regulation of neurotransmitters and their receptors, modulation of the HPA axis, control of inflammatory responses, management of oxidative stress, modulation of neurotrophic factors, regulation of gut microbiota and tryptophan metabolism, as well as other pathways.

In the experimental investigation of natural plant polysaccharide antidepressants, researchers conducted studies using various stress models including chronic unpredictable mild stress, social isolation-induced stress, social defeat stress, post-traumatic stress disorder, and acute behavioral despair depression. It is important to note that many depressed patients also suffer from comorbid psychiatric disorders such as obsessive-compulsive disorder and anxiety disorder. Therefore, future studies should examine the efficacy of polysaccharides in comorbidity models with these factors and explore combination therapy to enhance or optimize depression treatment.

Currently, mice and rats are the most commonly utilized models for depression research and testing of potential depression protective agents. Rodent models offer significant advantages over other mammals due to their metabolic and disease characteristics that closely resemble those of humans. Additionally, their smaller size, docile temperament, and ease of husbandry make them highly suitable for experimental purposes. However, it is important to acknowledge that rodents differ from mammals in terms of brain and neural structural organization; therefore, future studies should pay more attention to the specificities associated with rodent models. The higher complexity of human thinking and emotions compared to other species, as well as the similarity between non-human primate brains and those of humans in comparison to rodents, necessitate further preclinical studies involving higher animals to validate the alleviating and therapeutic effects of polysaccharides on depression. Ultimately, this will lead to real clinical trials involving human subjects.

Currently, numerous studies have substantiated the antidepressant effects of natural plant polysaccharides and elucidated their mechanisms of action from various perspectives. Furthermore, ongoing research continues to unveil additional insights into the antidepressant mechanisms of natural plant polysaccharides. As one of the primary bioactive constituents found in plants, natural plant polysaccharides exhibit a broad spectrum of biological activities. Therefore, conducting research on the antidepressant effects of natural plant polysaccharides holds immense significance for the development and utilization of natural plant-based polysaccharide antidepressant products.

Recently, there have been advancements in both preclinical and clinical research on the utilization of natural plant polysaccharides for depression treatment. A randomized, double-blind, placebo-controlled trial was conducted by experts to assess the effectiveness of LBP (*Lycium barbarum* polysaccharides, 300 mg/d for 6 weeks) in adolescents with subthreshold depression. The findings demonstrated that LBP effectively alleviated depressive symptoms such as cognitive impairments, retardation, and hopelessness in adolescents with subthreshold depression without any reported adverse events (Li et al., 2022). This experiment serves as a valuable point of reference for the application of natural plant polysaccharides at a human level. We eagerly anticipate further preclinical and clinical trials to validate the efficacy of natural plant polysaccharides in treating depression.

However, there are still numerous issues that require further exploration in the study of the antidepressant effects of natural plant polysaccharides. Firstly, the pathogenesis of depression is diverse and intricate, and natural plant polysaccharides predominantly exert their influence through multiple pathways, thereby rendering the mechanism behind their antidepressant properties even more complex. Although research on the antidepressant effect of polysaccharides has progressed from general observations to molecular-level investigations, our understanding of their signaling pathways and mechanisms remains insufficiently clear and comprehensive. Secondly, while cellular and animal studies on polysaccharides have increased significantly, clinical research in this area is relatively scarce. As research continues to advance, finding ways to extend the study of polysaccharide antidepressant effects to human subjects will remain a crucial point awaiting breakthroughs. Lastly, the physical properties, primary structure, advanced structure, and chemical modification of polysaccharides are closely linked to their biological activity; thus, investigating the conformational relationship between different natural plant polysaccharides with similar efficacy holds great significance for a thorough examination of their biological activity. However, research on the correlation between structure and antidepressant effects of natural plant polysaccharides is still in its early stages. With advancements in current technology for isolating and purifying polysaccharides as well as deepening studies into structural analysis over time, this may become a new avenue for future research on the antidepressant effects of natural plant polysaccharides.

Many active polysaccharides also play a significant role in the treatment of cardiovascular diseases in traditional Chinese medicine. However, most studies on the therapeutic potential of traditional Chinese medicine polysaccharides for depression are limited to animal and cellular models, which may not fully reflect their actual effects in humans. Currently, research on natural plant polysaccharides for depression treatment lacks depth and there is a scarcity of clinical trial studies, hindering widespread implementation in clinical settings and limiting their application within the field of medicine. The mechanism of action and signaling pathways of many natural plant polysaccharides for antidepressants remain unclear due to limitations in research methods, necessitating further investigation. Future studies should delve deeper into the mechanisms underlying natural plant polysaccharides’ ability to improve depression, clarifying previously studied but ambiguous mechanisms as well as exploring unreported ones. Additionally, future research should focus on clinical applications of polysaccharides, addressing gaps in knowledge such as tissue distribution and target specificity while also gathering more clinical evidence.

In conclusion, due to their unique advantages, polysaccharides derived from natural resources offer potential for investigating the mechanism of depression and serve as a valuable reference for further research on natural plant polysaccharides. Moreover, this exploration may lay the foundation for clinical applications. Furthermore, the development of antidepressant polysaccharides with minimal adverse reactions and high efficacy holds promise in expanding treatment options for depression.
